# The Gun Cotton and Paper

**Published:** 1847-01

**Authors:** 


*The Gun Cotton and Paper.* By M. Pelouze.—At the last meeting of the Academy of Sciences, M. Pelouze made some observations on the mode of preparation of his explosive paper; and stated, that two of his pupils had discovered an easy method of ascertaining whether or not the paper had been properly prepared. If the paper dissolves in sulphuric ether, it is perfect; and when it is not fit for use, it is insoluble. With the balistic and explosive properties of this new agent of destruction, by which is truly realized a maxim laid down by a celebrated French author—"Nothing in the universe is half so dangerous as a sheet of white paper"—and with the value of the discovery in the arts of war, we have nothing to do. M. Pelouze asserts, that by the action of nitric acid upon vegetable matter a compound is produced which consists only of cellulose and nitrogen, which resembling albumen, casein, and fibrin, by its elementary composition, might, like them, yield an alimentary substance. Experiments have been made upon three dogs; during four days each animal was fed on rice and explosive paper, taking every day nine oz. of rice and three of paper; it is true that the dogs did not lose weight during the period, but the exhibition of the rice must invalidate the experiment.—*Ibid.*

